# Evolution of Olfactory Functions on the Fire Ant Social Chromosome

**DOI:** 10.1093/gbe/evy204

**Published:** 2018-09-18

**Authors:** Amir B Cohanim, Etya Amsalem, Rana Saad, DeWayne Shoemaker, Eyal Privman

**Affiliations:** 1 Department of Evolutionary and Environmental Biology, Institute of Evolution, University of Haifa, Israel; 2 Department of Entomology, Huck Institutes of the Life Sciences, Pennsylvania State University; 3 Department of Entomology and Plant Pathology, University of Tennessee

**Keywords:** genome evolution, supergene, social evolution, social insects, odorant receptors

## Abstract

Understanding the molecular evolutionary basis of social behavior is a major challenge in evolutionary biology. Social insects evolved a complex language of chemical signals to coordinate thousands of individuals. In the fire ant *Solenopsis invicta*, chemical signals are involved in the determination of a polymorphic social organization. Single-queen (monogyne) or multiqueen (polygyne) social structure is determined by the “social chromosome,” a nonrecombining region containing ∼504 genes with two distinct haplotypes, SB and Sb. Monogyne queens are always SBB, while polygyne queens are always SBb. Workers discriminate monogyne from polygyne queens based on olfactory cues. Here, we took an evolutionary genomics approach to search for candidate genes in the social chromosome that could be responsible for this discrimination. We compared the SB and Sb haplotypes and analyzed the evolutionary rates since their divergence. Notably, we identified a cluster of 23 odorant receptors in the nonrecombining region of the social chromosome that stands out in terms of nonsynonymous changes in both haplotypes. The cluster includes twelve genes formed by recent *Solenopsis*-specific duplications. We found evidence for positive selection on several tree branches and significant differences between the SB and Sb haplotypes of these genes. The most dramatic difference is the complete deletion of two of these genes in Sb. These results suggest that the evolution of polygyne social organization involved adaptations in olfactory genes and opens the way for functional studies of the molecular mechanisms underlying social behavior.

## Introduction

Robinson et al. (2005) stated that “the goal of sociogenomics is to achieve a comprehensive understanding of social life in molecular terms,” thereby explaining the major evolutionary transition from solitary to social life (Szathmary and Smith 1995). The evolution of social behavior may involve both genetic and mimetic factors—a combination of nature and nurture. Identifying the genetic determinants of behavior in humans and other mammals is challenging due to the prominent component of learned behaviors (Plomin 1994; Ridley 2003). Conversely, the behavior of social insects is presumably determined largely by genetic effects (Smith et al. 2008). They display rather stereotyped behaviors, which can be more easily assayed (Robinson et al. 1997). This makes them a powerful model studying the molecular genetic basis of social evolution. Social behavior requires the fundamental functions of recognition of fellow colony members, communication, and coordination. In the social insects, chemical signaling by pheromones is a major mode of communication (Blomquist and Bagneres 2010). Therefore, the genes underlying the synthesis and perception of pheromones are of interest, especially their evolution and adaptive value as reflected through natural selection.

The fire ant *Solenopsis invicta* is a powerful study system where a supergene on the so-called “social chromosome” (Wang et al. 2013) determines whether a colony is monogyne (i.e., containing a single reproducing queen) or polygyne (i.e., containing multiple reproducing queens). The supergene is a 13-Mb long, nonrecombining region with two distinct haplotypes, SB and Sb, which has many of the properties of X–Y sex chromosome systems. Monogyne queens always carry an SBB genotype, while polygyne queens are always SBb. Sbb queens never reach reproductive maturity.

Behavioral experiments demonstrated that polygyne workers discriminate and accept only SBb queens based on olfactory cues (Keller and Ross 1998; Trible and Ross 2016). SBB queens are executed by polygyne workers, with attacks being predominantly led by SBb workers. The chemical cues responsible for queen discrimination are expected to be nonvolatile, contact-pheromones. Several long-chain hydrocarbons were found to be differentially expressed on the cuticle of the two queen genotypes (Eliyahu et al. 2011). Differential levels of hydrocarbons could signal the queen genotype quantitatively rather than qualitatively. Such hydrocarbons are generally considered prime candidates for pheromones responsible for recognition in social insects (Blomquist and Bagneres 2010).

The genetic basis for this social polymorphism was first assigned to two polymorphic loci: *pgm-3* and *gp-9* (Ross 1992, 1997; Keller and Ross 1998; Ross and Keller 1998). The first studies discovered this polymorphism in introduced populations of *S. invicta* in the United States, and subsequent studies found polymorphism also in the native range in South America, and in five additional closely related *Solenopsis* fire ant species, but not in other species of this genus (Krieger and Ross 2002, 2005; Gotzek et al. 2007). Thus, polymorphism of the social chromosome recently evolved in this clade. *gp-9* was found to encode an odorant binding protein (OBP), leading to the hypothesis that it could be involved in the olfactory recognition of queens (Krieger and Ross 2002; Gotzek et al. 2007). Multiple amino acid differences between the *B* and *b* alleles of *gp-9* were found, indicative of positive selection and supporting adaptive evolution of a novel olfactory function (Krieger and Ross 2002; Gotzek and Ross 2007). Nevertheless, other genes on the social chromosome could also play a role in olfactory functions.

Odorant receptors (ORs) are a large gene family that dramatically expanded in ants to over 400 genes, the highest number in any studied insect (Smith CD et al. 2011; Smith CR et al. 2011; Zhou et al. 2012; Roux et al. 2014; Zhou et al. 2015). Several subfamilies of ORs expanded dramatically in ants, particularly the so-called “nine-exon clade,” and several studies have inferred widespread positive selection in many branches of the OR gene tree (Roux et al. 2014; Engsontia et al. 2015; Zhou et al. 2015). ORs are expressed in olfactory sensory neurons where each receptor specifically recognizes an odorant molecule (Clyne et al. 1999; Hallem et al. 2004). Several studies implicated ant ORs in the perception of hydrocarbon pheromones (Ozaki et al. 2005; Sharma et al. 2015; McKenzie et al. 2016; Slone et al. 2017), including a study in *S. invicta* (Renthal et al. 2003). Some studies suggested that the dramatically expanded nine-exon OR clade is responsible for hydrocarbon perception (McKenzie et al. 2016), yet others reported that responses to hydrocarbons are not limited to this clade (Slone et al. 2017).

Here, we show that out of 504 genes on the social chromosome ORs display some of the most dramatic differences between the SB and Sb haplotypes. Comparative analyses of multiple genomes of fire ants and their close relatives identified a cluster of 23 tandem-duplicated ORs on the social chromosome. We reconstructed their evolution and found multiple recent gene duplications in the *Solenopsis* lineage. Molecular evolutionary and population genetic analyses show that these duplications were followed by episodic positive selection, especially in the putative ligand-binding site and the intracellular domain of the receptors. Most notably, a large deletion mutation on the Sb haplotype knocked out two of these *Solenopsis*-specific OR genes, implicating them in the differences between worker genotypes in their olfactory response to queen pheromones.

## Results

### Divergence and Gene Losses in the Social Chromosome

**Fig. 1.**
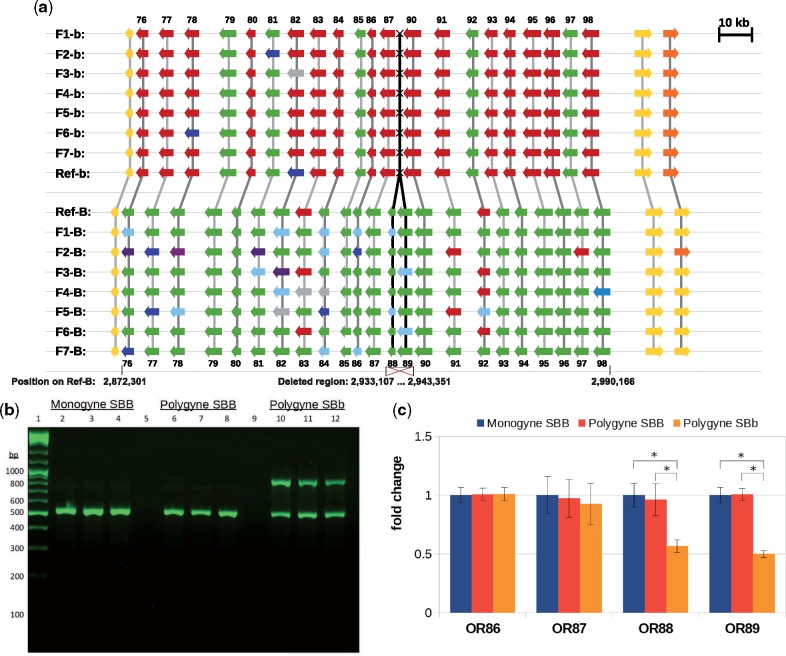
—(*a*) The cluster of the 23 *SiOR* genes on the social chromosome (*SiOR76-98*) and the deletion of a 10,258-bp long region in Sb containing *SiOR88* and *SiOR89*. The deletion was consistently found in eight independent de novo assemblies of whole-genome sequencing data from eight Sb males (F1-b to F7-b and the reference Sb genome sequence), relative to eight SB males (F1-B to F7-B and the reference SB). Colors represent distinct protein isoforms, with green marking the most frequent allele in SB, red the second most frequent allele, and other alleles in shades of cyan and blue. Grey indicates genes that were partially unassembled. Yellow and orange mark flanking non-OR genes, shown to allow comparison of synteny among the genomes. Genes (arrows) and their relative positions are drawn to scale. Genomic coordinates indicate positions on scaffold00008 of the SB reference genome. (*b* and *c*) Validation of the deletion of *SiOR88* and *SiOR89* in the Sb haplotype. Ten SBB workers were sampled from five monogyne colonies. Ten SBB workers and ten SBb were sampled from five polygyne colonies. (*b*) Banding pattern on 1.5% agarose gel after PCR of genomic DNA from SBB genotypes from monogyne (columns 2–4) and polygyne colonies (columns 6–8), and SBb genotypes from polygyne colonies (columns 10–12). Three representatives are shown of ten samples per group. The 875-bp amplicon encompasses the deletion in the Sb haplotype (primers at positions 2932440–2943560 on the SB genome). The 545-bp amplicon is outside the deleted region, so it is present in both SB and Sb (primers at positions 2753162–2753683 on the SB genome). (*c*) Relative amounts of genomic DNA of the four genes *SiOR86-89* in triplicates of workers from each of the same three groups (mean and standard error of 9, 10, and 10 samples per group, respectively). Asterisks indicate statistical significance at *P *<* *0.05 in a two-sided Tukey post hoc test following a mixed effects model analysis (see Materials and Methods).

We compared the SB and Sb haplotypes to investigate the evolution of the social chromosome, using the reference assemblies of SB and Sb, and whole-genome sequences of 14 additional haploid males from the introduced range in the United States (7 SB and 7 Sb). The nonrecombining region of the SB haplotype contains 504 putative protein-coding genes, excluding transposable elements. The most striking difference between the two haplotypes are two ORs that are present on the SB chromosome but completely missing from Sb, *SiOR88*, and *SiOR89*. The deletion encompasses 10,258 bp in the SB reference genome assembly. All other 502 genes are present in both SB and Sb. *SiOR88* and *SiOR89* are part of a cluster of 23 ORs (fig. 1*a*). All 16 genomes analyzed consistently show that the two genes *SiOR88* and *SiOR89* are present in the SB haplotype yet are missing in the Sb haplotype. The reference genome assembly shows all 23 ORs arranged head-to-tail in one genomic scaffold, but these genes were located in multiple separate scaffolds in some of the assemblies of the 14 samples that only had low coverage sequencing. Nevertheless, *SiOR88* and *SiOR89* were clearly identified in all seven SB assemblies, and in all but one of Sb assemblies (F2-b) the *SiOR87* locus was located in a scaffold that also contained the sequence on the other side of the deletion (the intergenic region next to *SiOR90*). We examined the other chromosomes of the fire ant to assess whether such deletions can also be found in other OR clusters. Apparent deletions may be due to incomplete assembly, so missing genes were considered as reliable deletion events only if neighboring genes on either side of the deletion were identified on the same genomic scaffold. Of the 16 genomes, only one Sb genome contained an apparent deletion of three genes (*SiOR42-44*) on chromosome 5.

The deletion of the social chromosome region harboring *SiOR88* and *SiOR89* was validated by two PCR approaches comparing heterozygous SBb workers and homozygous SBB workers from both monogyne and polygyne colonies. First, the region surrounding the site of the deletion in Sb was amplified by PCR. Samples of genotype SBb presented the amplicon of expected size (875 bp), whereas SBB samples did not (fig. 1*b*). As expected, PCR failed to amplify an amplicon from the SB haplotype where the genomic sequence between this pair of primers is over 10-kb long, as it contains the *SiOR88* and *SiOR89* loci. Second, genomic DNA of the loci *SiOR88* and *SiOR89* was quantified by real-time PCR (qRT-PCR). This analysis revealed that samples of genotype SBb display significantly lower relative amounts of *SiOR89* (by 50%) and marginally significant for *SiOR88* (by 37%), as compared with SBB samples from either monogyne or polygyne colonies (fig. 1*c*;*P *<* *0.0001 and *P *=* *0.07, respectively, Tukey post hoc tests following a mixed effects model analysis). SBB workers from polygyne and monogyne colonies showed similar relative amounts of genomic DNA for *SiOR88* and *SiOR89* and no significant differences were observed between the worker groups for *SiOR86* and *SiOR87*, two adjacent *SiOR*s found in both SB and Sb. These results fit the expectation of SBb individuals carrying only one copy of *SiOR88* and *SiOR89* on their SB chromosome and not on their Sb chromosome, whereas SBB individuals carry two copies.

### Selection Pressures on the Social Chromosome

To investigate selection pressures acting on protein-coding genes in the social chromosome, the genomic sequences of the two *S. invicta* haplotypes were compared with a draft genome assembly of the pharaoh ant *Monomorium pharaonis*, a closely related species in the tribe Solenopsidini. We were able to identify one-to-one orthologous genes in Sb and *M. pharaonis* for 331 out of the 504 putative protein-coding genes in the nonrecombining region of SB. Protein-coding sequences of *M. pharaonis* are 89% identical to *S. invicta* on an average, which allows accurate inference of the number of synonymous (d*S*) and nonsynonymous (d*N*) substitutions in each gene along the SB and Sb branches using the Nei and Gojobori method. The d*N*/d*S* ratio, averaged over the 331 genes, was 0.099 along the ancestral *S. invicta* branch, 0.087 along the SB branch, and 0.209 along the Sb branch. The difference between the SB and Sb branches is highly statistically significant (Fisher’s exact test *P *=* *0.0002). This higher evolutionary rate may be due to the lack of recombination of the Sb chromosome since its divergence from SB, which is expected to reduce the effectiveness of natural selection to remove slightly deleterious mutations. The high d*N*/d*S* ratio can also be interpreted as a combination of reduced purifying selection and positive selection pressure on a subset of social chromosome genes.

A very small number of substitutions per gene differentiate the SB and Sb haplotypes, which does not allow for accurate estimation of the d*N*/d*S* ratio per gene along the SB and Sb branches. In protein coding sequences, we found an average of 0.76 substitutions per gene in the SB branch and 1.22 substitutions in the Sb branch. 214 (64.7%) of the genes had zero substitutions in the SB branch and 158 (47.7%) in the Sb branch. Nonsynonymous substitutions were found in 33 genes (10.0%) on the SB branch and 88 genes (26.7%) on the Sb branch (supplementary table S1, Supplementary Material online). Five genes had nonsynonymous substitutions for both the SB and Sb haplotypes: a putative heparanase gene with 4 and 8 nonsynonymous substitutions on the SB branch and Sb branch, respectively; a glucose dehydrogenase with 3 and 4; a glutamate receptor delta-2 subunit alpha with 1 and 1, odorant receptor *SiOR80* with 1 and 1, and odorant binding protein SiOBP12 with 1 and 1 (nomenclature following Gotzek et al. 2011). The genes with nonsynonymous substitutions in the Sb branch include seven ORs, while those in the SB branch include two ORs (*SiOR80* appears in both lists). Additional genes with nonsynonymous substitutions in Sb may be involved in chemical communication and regulation of behavior: a putative sodium potassium calcium exchanger, an ejaculatory bulb-specific protein, a cuticular protein, and an octopamine receptor.

### Duplication and Adaptive Evolution of Odorant Receptors

The numerous OR genes identified in our analyses prompted us to focus on this gene family. The automatic gene annotation for *S. invicta* (Si_gnG; NCBI accession GCF_000188075.1) had only 182 putative OR genes. This is substantially fewer than the numbers found in the literature for other ants, which range between 291 to 407 ORs (Smith CD et al. 2011; Smith CR et al. 2011; Zhou et al. 2012). To address this possible discrepancy, we reannotated *S. invicta* ORs using a BLAST-based method, with ORs of four well annotated (OR-wise) ant genomes as search queries (see Materials and Methods), yielding 472 putative *SiOR* genes, including partial genes and possible pseudogenes. Using the same approach, we also identified 248 MpORs in the draft genome assembly of *M. pharaonis*. The predicted amino acid sequences were used to reconstruct the OR gene tree for the ant species *S. invicta*, *M. pharaonis*, *Pogonomyrmex barbatus*, and *Camponotus floridanus*.

**Fig. 2.**
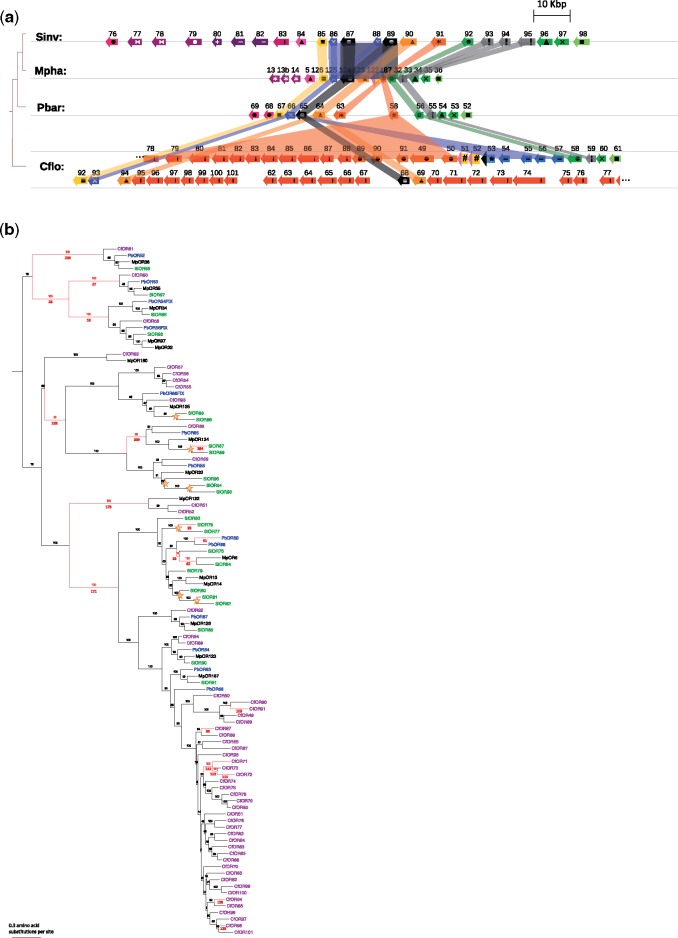
—(*a*) The cluster of 23 ORs on the *Solenopsis invicta* social chromosome and the orthologous clusters in *Monomorium pharaonis*, *Pogonomyrmex barbatus*, and *Camponotus floridanus*. Coloring and shading reflect orthology and gene duplications inferred from the gene tree presented in (b). Colors were chosen to indicate subgroups of closely related paralogs. Genes (arrows) and their relative genomic positions are drawn to scale. ORs were numbered independently in each species. In *C. floridanus*, the cluster is presented on two rows because of space limitations of the figure. (*b*) Subtree of the ant OR gene tree, showing the subfamily of ORs that includes the tandem cluster of 23 ORs located in the *S. invicta* social chromosome (gene names color coded by species). Maximum likelihood phylogeny reconstructed using RAxML, with bootstrap support shown above branches and branch numbers below. Stars mark Solenopsis-specific duplications. Branches in red indicate positive results in the branch-site test for positive selection (after FDR correction with *q *<* *0.1).

The nonrecombining region of the SB haplotype contains a cluster of 23 head-to-tail OR genes (*SiOR76-98*), all belonging to one OR subfamily, and a smaller cluster of five additional ORs belonging to a different subfamily (*SiOR462-466*). Figure 2*a* shows the cluster of 23 ORs in *S. invicta* and their homologs in the genomes of *M. pharaonis, P. barbatus*, and *C. floridanus*. The subtree of the gene tree corresponding to this cluster is presented in figure 2*b*. In each ant genome, this entire subfamily is arranged as a cluster of head-to-tail, adjacent paralogs, implying their evolution by tandem gene duplications. The gene tree allows approximate relative dating of many gene duplications that occurred in this subfamily during the evolution of ants: 14 duplications occurred in the ancestral lineage of the included species, one duplication is specific to Myrmicinae (after divergence of the *C. floridanus* lineage), at least one is specific to the tribe Solenopsidini (ancestral lineage of *M. pharaonis* and *S. invicta*), and many more are lineage-specific (most notably in the *C. floridanus* lineage). Seven gene duplication events (marked by stars in fig. 2*b*) occurred following the divergence of *Solenopsis*, leading to 12 *Solenopsis*-specific genes in three pairs and two triplets of paralogs: *SiOR77*, 78; *SiOR80*, 81, 82; *SiOR86*, 88; *SiOR87*, 89; and *SiOR93*, 94, 95 (fig. 2*b*). Note that the tandem pair *SiOR86*, 87 is paralogous to the tandem pair *SiOR88*, 89, which suggests that two adjacent genes were duplicated in a single tandem duplication event. This duplication, creating the four paralogs, is a recent *Solenopsis-*specific event, whereas the previous duplication that created the pair predates the divergence of the four ant species. The upstream pair, *SiOR88*, 89, is the same gene pair that is missing from the Sb haplotype (fig. 1). This suggests that *SiOR88*, 89 were present in the ancestor of SB and Sb, and were subsequently deleted in Sb.

We inferred selection pressures in this gene tree using the branch-site test of positive selection (Zhang et al. 2005). Branches colored red in figure 2*b* indicate positive selection (d*N*/d*S* >1) after correction for multiple testing (*q* value <0.1). Two gene duplications followed by positive selection pressures can be observed in branches 236, 28, 27, 18 (*q *=* *0.014, 0.014, 0.016, 0.014, respectively). These gene duplications preceded the speciation of the four ant species included in this analysis. Positive selection also occurred on branch 228 (*q *=* *0.096), which represents the ancestor of *SiOR86*–89, 93–95. Following a gene duplication within this subtree, positive selection is observed on branch 209 (*q *=* *0.020), which is the ancestor of *SiOR87*, 89. A subsequent *Solenopsis*-specific gene duplication produced *SiOR87* and *SiOR89*, followed by positive selection on the *SiOR87* gene (branch 204, *q *=* *0.053). These results unravel recurrent duplication and positive selection pressures on this subset of paralogs in the *SiOR* gene cluster, suggesting continued adaptive evolution in the fire ant lineage and the evolution of novel olfactory functions in these genes.

### Polymorphism in Social Chromosome Odorant Receptors

The protein-coding sequences of 22 of the 23 *SiOR* genes differ between the SB and Sb haplotypes. We used the abovementioned 16 whole genome sequences of *S. invicta* males (8 SB and 8 Sb) to identify polymorphic sites both between and within the two haplotype groups (supplementary table S2, Supplementary Material online). This analysis revealed that 14 *SiOR*s have nonsynonymous polymorphisms within the sample of SB haplotypes, while only one (*SiOR87*) has nonsynonymous polymorphisms within the Sb sample. Fourteen ORs have fixed nonsynonymous differences between SB and Sb, including *SiOR86* and *SiOR87* (paralogs of the deleted *SiOR88* and *SiOR89*). Relative to the ancestral sequence, 39% (9 out of 23) of the ORs had fixed nonsynonymous substitutions in SB compared with 10% of all genes on the social chromosome (supplementary table S1*a*, Supplementary Material online). In Sb, 57% of ORs (12 out of 21) had fixed nonsynonymous substitutions compared with 27% of all genes (supplementary table S1*b*, Supplementary Material online). This represents a significant enrichment of nonsynonymous substitutions in ORs (χ^2^ test *P* value <10^−5^ for SB, *P* value <10^−2^ for Sb). These higher proportions may be the result of positive selection, however, statistical tests using the d*N*/d*S* ratio cannot infer positive selection because of the small number of substitutions per gene on the SB and Sb branches. Nevertheless, we note that *SiOR78* shows evidence for positive selection (according to the branch-site test) in the ancestral branch after a recent *Solenopsis-*specific duplication, as well as two nonsynonymous changes in the Sb branch (supplementary table S2, Supplementary Material online). *SiOR84* shows evidence for positive selection after its duplication (specific to the Solenopsidini tribe), as well as two nonsynonymous substitutions in the SB branch and two in the Sb branch. Altogether, these results suggest ongoing adaptive evolution in these genes.

### Evolutionary Change in the Context of Odorant Receptor Structure

**Fig. 3.**
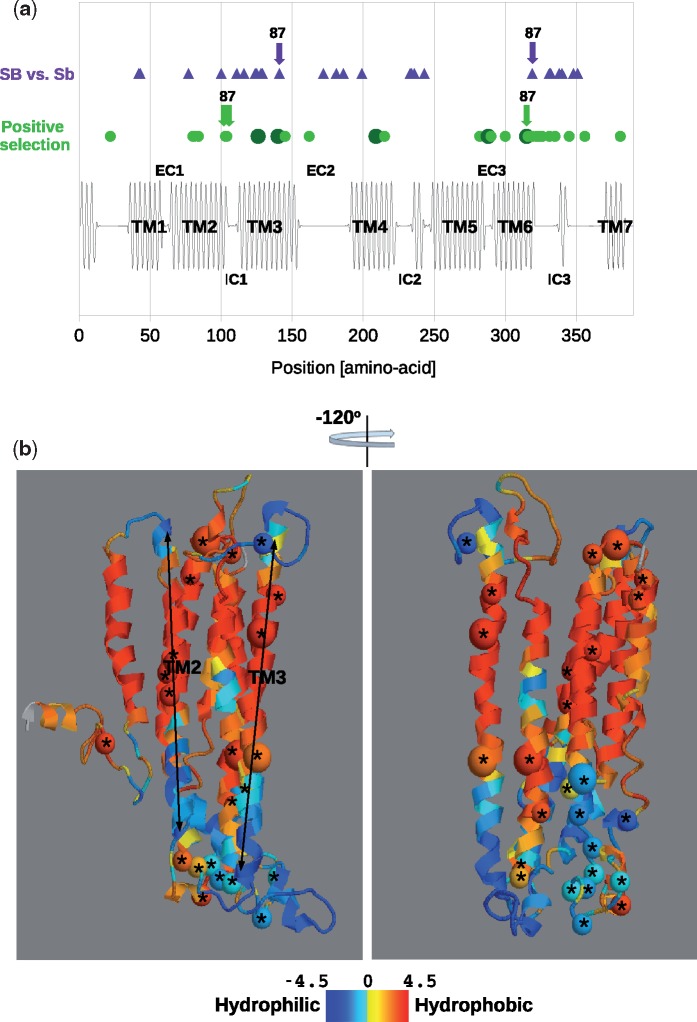
—Structural context of evolutionary changes in odorant receptors encoded by the social chromosome. (*a*) A linear display of alpha helix alternations, and approximation of the extracellular (EC), transmembrane (TM) and intracellular (IC) regions. Green circles depict sites that experienced positive selection according to the branch-site test along all branches of the gene tree (large circles represent sites where two branches were positive), while purple triangles depict amino acid differences between the SB and Sb haplotypes. Arrows point to sites in *SiOR87*. (*b*) Model of insect OR protein structure displaying the positive selected sites as spheres marked by asterisks (large spheres represent sites where two or more branches had positive selection), colored by the median hydrophobicity index of the 23 *SiOR* sequences, from shades of red (hydrophobic amino acids) to blue (hydrophilic).

Amino acid changes in the divergence of the 23 *SiOR*s from their corresponding ancestral sequences were investigated in the context of the predicted 3D protein structure of insect ORs (Hopf et al. 2015). These recent changes were compared with the more ancient amino acids substitutions that experienced positive selection according to the branch-site test in all branches of the gene tree of this subfamily (i.e., not specific to *Solenopsis*; see fig. 2*b*). This comparison highlights regions of the protein structure repeatedly modified during evolution. Figure 3*a* shows the distribution along the linear protein structure of sites that experienced positive selection in one or more branches of the gene tree (branch-site test posterior probability >0.9) and sites with amino acid differences between the SB and Sb alleles. Multiple sites of both groups mapped to the large intracellular loop domain IC3 and to the transmembrane domain TM3. Ten positively selected sites are located in the IC3 domain and five positively selected sites are located in the TM3 domain (FDR corrected binomial test *q* value = 0.0003 for 10 out of 33 sites to be found in a 24 amino acid region out of a total sequence length of 390 for IC3; *q* value = 0.29 for 5 sites to be found in a 25 amino acid region for TM3; full results in supplementary table S4, Supplementary Material online). The functional effect of amino-acid differences between the SB and Sb haplotypes (purple triangles in fig. 3*a*) were evaluated by classifying them by LALIGN into conservative or not (see Materials and Methods). Eleven of the 26 substitutions the 21 ORs are nonconservative, including five of the seven positions in IC3 (supplementary table S5, Supplementary Material online). *SiOR87* is noteworthy because of multiple sites with positive selection and haplotype differences (marked by arrows), located in these protein domains. Figure 3*b* shows the distribution of the positively selected sites on a 3D model of the OR protein structure, with color-coding of amino acid hydrophobicity. The left side view shows several sites along the hydrophobic regions of TM2 and TM3, while the right-side view shows the cluster of sites in the more hydrophilic IC3 domain.

## Discussion

This study identified candidate genes implicated in the evolution of social polymorphism by surveying the fire ant’s social chromosome for evidence of adaptive evolution. Comparative genomic analyses of the differences between SB and Sb identified a loss of two genes on the Sb haplotype, as well as a higher rate of amino acid substitution in this haplotype. The higher evolutionary rate is in line with previous observations that the Sb haplotype is accumulating more deleterious mutations, analogous to the dynamics of Y chromosome evolution, and that there is much lower nucleotide diversity in the Sb haplotype group (Wang et al. 2013; Pracana, Levantis, et al. 2017). These observations may be interpreted as reflecting reduced purifying selection. The elevated evolutionary rate may also be the result of positive selection pressure on a subset of genes, as part of the adaptive evolution of traits related to the derived polygyne social form. Notably, the fastest evolving genes include eight ORs and one OBP (supplementary table S1, Supplementary Material online), in addition to the previously described OBP known as *gp-9*. These results thus suggest recent adaptations in olfactory functions, potentially related to chemical signaling by pheromones involved in the regulation of social organization.

Other genes with haplotype-specific amino acid substitutions have various predicted molecular functions including enzymatic, metabolic, and regulatory functions. Notably, several of the fastest evolving genes have predicted neurological functions: a glutamate receptor, an octopamine receptor, and an ion transporter. The octopamine receptor is especially noteworthy because octopamine is a major neurohormone, neuromodulator, and neurotransmitter that mediates many behavioral and physiological responses in insects (David and Coulon 1985; Roeder 1999). Studies in the honeybee *Apis mellifera* found that octopamine mediates olfactory learning and the formation of olfactory memory (Hammer 1993; Hammer and Menzel 1998). Octopamine is also related to dominance and aggressive behavior in the bumblebee *Bombus terrestris* (Bloch et al. 2000) and the queenless ant *Streblognathus peetersi* (Cuvillier-Hot and Lenoir 2006). The octopamine receptor gene found on the social chromosome is highly conserved across insects, with the *S. invicta* protein presenting 86% amino acid sequence identity to the honeybee ortholog. Therefore, the observation of a high evolutionary rate in the fire ant Sb haplotype of this gene (four amino acid substitutions) suggests adaptive evolution related to olfactory perception, potentially related to the chemical signaling mediating social organization in fire ants.

The most notable difference between the SB and the Sb haplotypes is the deletion of two OR genes in Sb. These genes are completely missing from all eight Sb males that were sequenced by whole genome sequencing, and the deletion was also confirmed by PCR in ten samples of SBb workers. Thus, the frequency of the deletion among Sb haplotypes is >99.7% (95% confidence interval assuming a binomial distribution with *n* = 18). Our samples represent the introduced *S. invicta* populations in the United States. It would be interesting to also investigate native *S. invicta* populations for the presence of this deletion. The US population has experienced a severe bottleneck during the introduction. Estimates of the effective population size of the founder population range between 10 and 30 singly mated queens (Ross and Shoemaker 2008; Pnina Cohen and Eyal Privman, unpublished data), which corresponds to 15 and 45 unrelated alleles per locus. Thus, it is possible that there are Sb haplotypes without the deletion in the native range, which did not pass through the population bottleneck. However, SB and Sb diverged hundreds of thousands of years ago and are no longer recombining (Wang et al. 2013), so the deletion may have been fixed already in the native range. This question would require additional investigation of native populations.

The two deleted genes *SiOR88* and *SiOR89* are part of a cluster of 23 ant-specific ORs. Interestingly, this OR subfamily is outside of the so-called nine-exon clade, which was suggested to be responsible for the perception of cuticular hydrocarbons (McKenzie et al. 2016). However, a more recent study reported that responses to hydrocarbons are not limited to this clade (Slone et al. 2017). We propose that the social chromosome OR cluster contributed to adaptive evolution in fire ants based on the inference of recent expansion in this subfamily in the *Solenopsis* lineage, followed by positive selection. Twelve of the 23 genes arose from recent duplications in the *Solenopsis* genus. Interestingly, this cluster was found to be one of the less dynamic OR subfamilies in ants in terms of gene gain and loss, which makes the dramatic expansion in *Solenopsis* even more significant (see Supplementary Figure S2 by Zhou et al. 2015). The two gene pairs *SiOR86*, *87* and *SiOR88*, *89* are the result of a recent tandem duplication in the *Solenopsis* lineage, and *SiOR88*, *89* were subsequently deleted in the Sb haplotype. We have provided evidence for positive selection on three of the twelve *Solenopsis*-specific genes following their duplication. *SiOR87*, the paralog of *SiOR89*, stands out in terms of both positive selection in the *Solenopsis* lineage and divergence between SB and Sb. Interestingly, the ortholog of *SiOR87* and *SiOR89* in the carpenter ant *Camponotus floridanus* (CfOR68) is one of the few ORs that is significantly overexpressed in minor versus major workers, and the only one in this OR subfamily (Dataset S5 of Zhou et al. 2012). Thus, the function of this receptor may be related to behavioral differences and task allocation, such as the tendency of minor workers to fulfill nursing tasks (Zube and Rossler 2008).

The evidence for adaptive evolution of social chromosome ORs parallels the evolution of OBPs. The two highly divergent alleles of the OBP *gp-9* were the first polymorphism discovered to be completely linked with the social polymorphism (Ross 1997; Keller and Ross 1998; Ross and Keller 1998). Later genomic studies discovered at least eight more OBPs in the nonrecombining region linked to *gp-9* (Gotzek et al. 2011; Pracana, Priyam, et al. 2017). Pracana, Priyam, et al. (2017) report a tenth OBP in Sb, which is not found in SB. This appears to be a recent duplication of SiOBP12. The deletion of the two ORs together with the duplication of an OBP in Sb reinforce the hypothesis that the most dramatic mutations in Sb underlie the evolution of olfactory functions in polygyne colonies, which makes these genes prime candidates for queen discrimination by workers.

Altogether, these results are in line with adaptive evolution by gene duplication and neofunctionalization or subfunctionalization, that is, the evolution of novel functions or the specialization of different paralogs in distinct subfunctions of the ancestral copy. Previous studies in insects and vertebrates suggested that ORs evolve by a rapid birth-and-death process, allowing for adaptation of olfactory functions (Niimura and Nei 2006; Zhou et al. 2012). The commonly proposed model involves divergent sequence evolution following gene duplication, which generates new paralogs with derived functions (Ohno 1970). For example, newly derived OR paralogs may evolve different ligand specificities. We propose that dynamic gene gain/loss evolution altered the functions of this receptor subfamily in fire ants, allowing them to fine tune and elaborate the olfactory response to a set of olfactory cues, such as queen pheromones. The derived receptors may have evolved specificity to new ligands, novel regulatory mechanisms of gene expression, and/or differences in signal transduction from the receptors to intracellular pathways. Thus, these genes are prime candidates for understanding the evolution of social structure in *Solenopsis* fire ants.

Projection of the results from the evolutionary analysis onto the protein structure highlighted two domains as the targets of positive selection during the long-term evolution of this OR subfamily in ants: the transmembrane TM3 domain and the intracellular IC3 domain. These domains also contain multiple recent amino acid changes in SB and Sb. The TM3 domain of a *Drosophila* OR was implicated in ligand-binding activity (Nichols and Luetje 2010). This is also a highly hydrophobic domain that could bind a hydrophobic ligand, which is in line with the hydrophobic nature of a set of hydrocarbons that were shown to be differentially expressed on the cuticle of fire ant queens from the two social chromosome genotypes (Eliyahu et al. 2011). Therefore, adaptation in this domain may be responsible for altered ligand specificity, both before and after the formation of the SB and Sb haplotypes. We propose that this subfamily of ORs originally evolved the function of queen pheromone recognition in ancestral ant species, and were more recently co-opted for the function of queen discrimination in the socially polymorphic fire ants. The IC3 loop domain is much less hydrophobic, and faces the intracellular side of the membrane. Thus, adaptation in this domain may be involved in signal transduction from the receptor to intracellular factors.

Several models were proposed to explain the regulation of queen number and queen genotype discrimination by fire ant workers (Fletcher and Blum 1983; Keller and Ross 1999; Gotzek and Ross 2007). Fletcher and Blum (1983) first suggested that different quantities of a queen pheromone produced by different queens determines their acceptance by a nest’s worker population. Later studies showed that introduced SBB queens are recognized and executed by polygyne workers, and that SBb workers are overrepresented among the attackers (Keller and Ross 1998). Keller and Ross (1998) hypothesized that two pheromones explain the discrimination of queens by workers: a maturity pheromone signaling the reproductive state of the queen and a “green beard” pheromone signaling the presence of the Sb allele. The maturity pheromone triggers an aggressive response in workers, whereas the green beard pheromone leads to acceptance by workers in polygyne nests. Gotzek and Ross (2007) elaborated the model by suggesting the Sb allele is responsible for two effects: signaling its presence by the green beard pheromone and repression of signaling by the maturity pheromone. The *b* versus *B* alleles of the odorant binding protein GP-9 were suggested as the mechanistic basis for several of these proposed models. For example, amino acid substitutions between the two alleles may have changed the ligand-binding affinity of GP-9, which could alter the olfactory response of workers to different quantities of the putative queen pheromone.

Differential expression of ORs in workers could play a part in the mechanistic basis for these models, in the same manner as previously proposed for GP-9. Higher expression of a receptor is predicted to result in a lower response threshold for its cognate ligand, and may also affect the firing rate of olfactory sensory neurons (Grant et al. 1995; Duchamp-Viret et al. 1999). In monogyne colonies, workers respond to a pheromone signaling the reproductive status of queens, which induces workers to kill any reproductive queen introduced to the colony. Polygyne colonies are generally less aggressive toward nonnestmates, and they discriminate between introduced queens based on their genotype. The acceptance of SBb queens was interpreted as a “green beard” effect—the Sb haplotype is a selfish allele whose carrier preferentially cooperates with other carriers of the same allele, which they recognize by some observable signal (Hamilton 1964a,b; Dawkins 1976; Keller and Ross 1998; Lehmann and Keller 2006; Gardner and West 2010). Thus, SBb queens may overexpress a green beard pheromone, relative to SBB queens (Keller and Ross 1998). We hypothesize that fire ant workers express an OR that responds to such a pheromone, leading to acceptance of a new queen, that is, suppressed aggression. One of the deleted ORs in the Sb haplotype could be responsible for this function by triggering an acceptance behavior in response to the green beard pheromone. The deletion in Sb is expected to result in approximately halving the quantity of these OR loci in polygyne SBb individuals (fig. 1*c*), which is expected to raise the olfactory response threshold. Thus, only SBb queens with a high level of the green beard pheromone would pass the acceptance threshold of these workers.

## Materials and Methods

### Genomes Assembly and Alignments

To investigate the evolution of genes in the social chromosome, genomic sequences of the two *S. invicta* haplotypes SB and Sb (Wurm et al. 2011; Wang et al. 2013) were compared with the draft genome of *M. pharaonis* (NCBI accession BBSX00000000.1), which we assembled using SOAPdenovo2 (Li et al. 2010). In addition to the *S. invicta* reference genome (version Si_gnH; NCBI accession AEAQ00000000), we also assembled genomic sequences of SB and Sb haploid son pairs from each of seven unrelated SB*/*Sb queens collected in Athens, Georgia (Wang et al. 2013) (NCBI accession SRP017317) using SOAPdenovo2. Multiple alignment of the genomes was inferred using Mugsy (Angiuoli and Salzberg 2011).

### Gene Annotation and Orthologous Gene-Sets

Gene annotation was conducted using genBlast (She et al. 2009; She et al. 2011) (http://genome.sfu.ca/genblast/; last accessed on Nov 2015), in which translated BLAST hits to exons were grouped to represent a putative gene models, while stitching hits at predicted splice site junctions. Predicted protein sequences from the *S. invicta* reference genome were used as queries (http://www.ncbi.nlm.nih.gov/genome/annotation_euk/Solenopsis_invicta/100/) to annotate the new *M. pharaonis* genome assembly. OR genes were annotated in *M. pharaonis* and *S. invicta* using previously annotated ORs of four ant species: 320 LhORs from *Linepithema humile* (Smith CD et al. 2011), 291 PbORs from *Pogonomyrmex barbatus* (Smith CR et al. 2011), 377 HsORs from *Harpegnathos saltator* (Zhou et al. 2012), and 407 CfORs from *Camponotus floridanus* (Zhou et al. 2012). Novel gene sequences are available in supplementary files S1–S4, Supplementary Material online, and also deposited in the figshare database (accession number: 3822126; https://dx.doi.org/10.6084/m9.figshare.3822126.v1). The OR gene cluster in the social chromosome was manually verified by alignment to orthologs in the previously annotated species (*P. barbatus*, *C. floridanus*, *L. humile*, and *H. saltator*) and by prediction of secondary structure by PRALINE (Simossis and Heringa 2005) to verify the completeness of the 7-transmembrane structure of the receptors. The assemblies of the 14 SB and Sb haploid males were annotated as described earlier, and screened for gene deletion or insertion events. These samples were sequenced at an average depth of ∼10×, which results in fragmented assemblies. Therefore, events were considered only if at least two neighboring genes were identified on either side of the deleted/inserted gene, thereby excluding apparent deletions close to the edges of scaffolds. Putative events were then manually inspected to verify the alignment and gene annotation.

OMA (http://omabrowser.org/oma/about/; last accessed on July 2015) was used to infer gene orthology between the closely related genomes SB, Sb, and *M. pharaonis*. The OMA algorithm starts with all-against-all alignments and then builds hierarchical orthologous groups (HOGs) according to the taxonomical hierarchy, such that orthologs descended from a single ancestral gene in a given taxonomic range are grouped together (Altenhoff et al. 2013, 2015). Thus, we obtained both one-to-one orthologous gene pairs and clusters that contain paralogous genes.

### Validation of Deletion in Sb

#### Samples

Five polygyne and five monogyne *S. invicta* colonies were collected in October–December 2015 in Gainesville, Florida. Colonies were maintained in the laboratory for two weeks postcollection in standard conditions (Jouvenaz et al. 1977). Workers were frozen on dry ice and kept in −80° C until further analysis. DNA samples were extracted from whole bodies of 130 randomly selected individual workers (20 and 6 individuals per each polygyne/monogyne colony, respectively) using the Allrep DNA/RNA Micro kit (Qiagen, Valencia, CA) according to the manufacturer’s instructions. Discrimination between the *gp-9^B^* and *gp-9^b^* alleles (corresponding to the SB and Sb haplotypes) was done using the Valles and Porter (2003) method. Homozygous SBB (39%) and heterozygous SBb (61%) but no homozygous Sbb workers were found in polygyne colonies. All workers found in monogyne colonies were homozygous SBB. Each ant was placed in an individually numbered tube that did not provide information about their genotype, colony of origin or social form. The information above was combined with DNA qRT-PCR measurements only after the analyses were completed.

#### Primer Design

Primers were designed using Primer-BLAST (http://www.ncbi.nlm.nih.gov/tools/primer-blast/; last accessed on April 2016). Two pairs of primers were designed to experimentally validate the deletion of *SiOR88*, 89 in the Sb haplotype. The first pair was designed to encompass the deletion, corresponding to 875 bp in the Sb haplotype and >10,000 bp in the SB haplotype. An additional pair of primers was designed outside of the deleted region, producing an identical product of 545 bp in both SB and Sb haplotypes. For qRT-PCR, four pairs of primers were designed for the genes located within the deletion (*SiOR88* and *SiOR89*) and in the two adjacent genes (*SiOR86* and *SiOR87*). To control for PCR efficiency and individual differences across samples, we relied on two housekeeping (HKG) genes: *rpl18* (NCBI accession EH413666) and *ef1-beta* (NCBI accession EH413796). We used newly designed primers to match the HKG annealing temperature with the other primers in this study. For the full list of primers see supplementary table S3, Supplementary Material online.

#### Experimental Validation of the Deletion in the Sb Haplotype

Experimental validation of the deletion of *SiOR88*, 89 was examined in 30 individual workers (10 monogyne SBB, 10 polygyne SBB and 10 polygyne SBb workers, sampled evenly from the five monogyne and five polygyne colonies) via multiplex PCR amplifications of genomic DNA using the following conditions: initial denaturation at 94°C for 2 min, 37 cycles of denaturation at 94°C for 30 s, annealing at primer specific temperature for 30 s, extension at 72°C for 30 s; followed by a final extension at 72°C for 5 min. PCR reaction mixtures consisted of 50 ng of DNA, 12.5 µl of PCR mastermix (Promega), 1 µM of each primer and water brought to the final volume of 25 µl. PCR products were electrophoresed in 1.5% agarose gel (Invitrogen) and detected with ethidium bromide. Fragments of DNA were visualized by a UV-Transilluminator (Bio-Rad).

#### Relative Quantification of Genomic DNA

29 individual worker samples (9 monogyne SBB, 10 polygyne SBB and 10 polygyne SBb workers, sampled evenly from the five monogyne and five polygyne colonies) were used to quantify the relative amounts of *SiOR* 86–89 genomic DNA (gDNA). The 10 different colonies used were equally represented in the samples. Relative amounts of gDNA were determined by qRT-PCR on an ABI Prism7900 sequence detector using the SYBR Green detection method. For each of the six primer pairs (four ORs and two HKGs) three technical replicates were run. For each of those, 2 μl of gDNA (20 ng/sample) were combined with 5-μl SYBR-Green (Bioline, Luckenwalde, Germany), 0.2 μl of each forward and reverse primer (10 µM stock) and 2.6 μl DEPC-water. Negative control samples were present on each plate. PCR product quality and specificity was verified by melt curve analysis. A standard curve was generated for each set of primers using five different concentrations of gDNA in order to determine *r*^2^ and efficiency. Triplicate reactions were performed for each of the samples and averaged for use in statistical analysis. To avoid artifacts due to inconsistent pipetting, samples with CT values >0.5 above the average CT of the three technical replicates were excluded. All 29 qRT-PCR samples were analyzed. These criteria were pre-established and are used in all of our studies. The relative amounts of gDNA for each gene were normalized to the geometric mean of two HKGs using the 2^−^^Δ^^*Ct*^ technique. For figure presentation, the relative amounts of gDNA were normalized relative to the monogyne SBB group.

#### Statistical Analysis

Statistical analyses were performed using JMP Pro version 12.1. Comparisons of log-transformed genomic DNA (gDNA) relative amounts were done using a mixed model with group (monogyne SBB, polygyne SBB or polygyne SBb) as fixed effect, relative amounts of gDNA as response and colony as a random factor. Significant differences were followed by a Tukey type post hoc test. The Shapiro–Wilk test rejected normality for *SiOR87*, 89 but not for *SiOR86*, 88. Thus, we tested for differences in concentrations of genomic DNA in *SiOR87*, 89 also using the nonparameteric Kruskal–Wallis test, which found comparable and significant results. Equivalence of variances was tested using O’Brien test, which did not reject the assumption of equivalence for all four genes (table 1).

**Table 1:** Statistical analysis of differential quantities of genomic DNA

	Nparm	DFNum	DFDen	*F* ratio	*P* value	
*SiOR86*	2	2	26	0.004	0.99	
*SiOR87*	2	2	12	0.09	0.90	
*SiOR88*	2	2	17.8	3.77	0.04	
*SiOR89*	2	2	5	64.02	0.0003	
Post hoc Tukey test for *SiOR88*:						

	**Difference**	**Std error**	**t Ratio**	***P* value**	**Lower 95%**	**Upper 95%**

Mono SBB vs poly SBB	−0.0006	0.03	−0.21	0.97	−0.09	0.07
Mono SBB vs poly SBb	−0.08	0.03	−2.44	0.06	−0.17	−0.003
Poly SBB vs poly SBb	−0.07	0.03	−2.38	0.07	−0.16	0.005
Post hoc Tukey test for *SiOR89*:						

	**Difference**	**Std error**	**t Ratio**	***P* value**	**Lower 95%**	**Upper 95%**

Mono SBB vs poly SBB	0.002	0.03	0.06	0.99	−0.12	0.12
Mono SBB vs poly SBb	−0.42	0.04	−10.07	0.0004	−0.56	−0.28
Poly SBB vs poly SBb	−0.42	0.03	−11.45	0.0002	−0.55	−0.3

### Phylogeny Reconstruction

The OR gene tree was reconstructed based on ORs from four ants: *S. invicta, M. pharaonis, P. barbatus*, and *C. floridanus*. Predicted amino acid sequences were aligned using MAFFT version 7, accurate variant E-INS-i (Katoh et al. 2005), with the default parameters: scoring matrix BLOSUM-62, gap opening penalty 1.53. This alignment was used to reconstruct the ant OR gene tree using RAxML version 8.1.15 (Stamatakis 2006) with the PROTCATLG model, and 100 bootstraps repeats. A subtree of 124 ORs contained the 23 social chromosome ORs (*SiOR76-98*). This subgroup of 124 sequences was realigned using GUIDANCE version 2.01 (Penn et al. 2010) with the aligner PAGAN (Loytynoja et al. 2012) as a codon alignment. Unreliably aligned residues were masked at 0.8 GUIDANCE score cutoff (i.e., low-scoring codons were replaced with “NNN”). Then the phylogenetic tree was rebuilt using RAxML. The phylogenetic tree of the social chromosome subtree is provided in Newick format in supplementary file S5, Supplementary Material online and was deposited in the figshare database (accession number: 3822126; https://dx.doi.org/10.6084/m9.figshare.3822126.v1).

### Tests for Positive Selection

The ratio of nonsynonymous to synonymous substitutions (d*N*/d*S*) was used to test for positive selection on OR genes. Each one-to-one orthologous gene-set from the four genome data set (described earlier) was used for testing three branches: the branch of the *S. invicta* lineage after the divergence from *M. pharaonis*, and the terminal branches of SB and Sb. Coding sequences of each orthologous gene-set, were aligned using PRANK (Loytynoja and Goldman 2008), and sites with alignment uncertainty were masked with a 0.8 score cutoff using GUIDANCE, based on HoT (Heads-Or-Tails) scores (Landan and Graur 2008; Penn et al. 2010).

Two d*N*/d*S*-based approaches were used to test for positive selection: the modified branch-site test model A (Zhang et al. 2005) and the Nei and Gojobori method (Nei and Gojobori 1986). The *S. invicta* branch was tested by the branch-site test implemented in the PAML package, version 4.8a (Yang 2007). This is a likelihood ratio test (LRT) that compares a model allowing positive selection on one of the branches of the phylogeny to a model that allows no positive selection. LRT *P* values for each branch were corrected into *q* values to control the false discovery rate (FDR) (Benjamini and Hochberg 1995). For each branch where the LRT was positive at FDR <10%, specific codons under positive selection were identified based on posterior probability >0.9 for d*N*/d*S* >1. The same approach was used to test each branch of the OR gene tree.

The terminal branches of SB and Sb were too short for the branch-site test (branch lengths of 0.00105 and 0.00153, respectively). Therefore we calculated d*N* and d*S* of these branches using custom code that implements the Nei and Gojobori method (Nei and Gojobori 1986; code deposited in the figshare repository: https://doi.org/10.6084/m9.figshare.6911945.v1): d*S* and d*N* were calculated by counts of synonymous and nonsynonymous substitutions per synonymous and nonsynonymous codon sites. In these terminal branches, it is not possible to calculate the d*N*/d*S* ratio per gene because many genes had zero synonymous substitutions. Instead we reported the list of top genes ranked according to their d*N* values, and examined the predicted molecular functions represented by this list.

### Odorant Receptor Structure Analysis

The results of the evolutionary analysis of the 23 ORs in the social chromosome cluster were examined in the context of their predicted 3D protein structure. The *SiOR* sequences were aligned against a model of *Drosophila melanogaster* OR85b, an *ab initio* structural prediction performed by Hopf et al. (2015) using the evolutionary couplings method (Marks et al. 2012). The *SiOR*s were aligned based on their amino acid sequence and predicted secondary structure by PRALINE (Simossis and Heringa 2005). This alignment was used to map amino acid positions under positive selection and differences between the SB and Sb haplotypes to the protein structure. The average hydrophobicity of amino acids in each position was calculated for the 23 *SiOR*s using the Kyte and Doolittle hydropathy index (Kyte and Doolittle 1982).

Amino-acid differences between the SB and Sb haplotypes were classified into conservative or not by LALIGN (https://embnet.vital-it.ch/software/LALIGN_form.html; last accessed February 2016) using the PAM 250. matrix (http://prowl.rockefeller.edu/aainfo/pam250.htm; last accessed February 2016) 

## Supplementary Material

Supplementary Data
