# Supplementary material for: Evolution of Olfactory Functions on the Fire Ant Social Chromosome
Source: Genome Biol Evol. 2018 Sep 18;10(11):2947–60. doi: 10.1093/gbe/evy204 (PMC6279166; doi:10.1093/gbe/evy204)
Supplement: Supplementary Data [file evy204_supp.zip › SUPPLEMENTARY MATERIAL Legends.docx]

# Supplementary Legends

**Table S1:** Synonymous and non-synonymous substitutions in the SB (a) and Sb (b) haplotypes of social chromosome genes

**Table S2:** Divergence and polymorphism in the cluster of *S. invicta* OR genes

**Table S3:** Primer list

**Table S4:** Binomial tests for positive selection enrichment in OR structural domains

**Table S5:** Conservative and non-conservative amino-acid differences between the SB and Sb haplotypes

**Files S1-S3:** Social chromosome genes annotated or re-annotated in this study (predicted cDNA and protein sequences in FASTA format, and annotation coordinates in GFF format)

**File S4:** OR genes annotated or re-annotated in this study in the entire genome of *S. invicta*

**File S5:** Phylogeny of the social chromosome clade of the OR gene tree
