# Supplementary material for: Evolution of Olfactory Functions on the Fire Ant Social Chromosome
Source: Genome Biol Evol. 2018 Sep 18;10(11):2947–60. doi: 10.1093/gbe/evy204 (PMC6279166; doi:10.1093/gbe/evy204)
Supplement: Supplementary Data [file evy204_supp.zip › Table S1.Synonymous and nonsynonymous substitutions in SB and Sb.gnH.pdf]

Table S1: Synonymous and nonsynonymous substitutions in the SB (a) and Sb (b) haplotypes of social chromosome genes

Table S1a: SB haplotype

Local gene ID (assembly Si_gnH)	NCBI accession number (assembly Si_gnG)	Top blast hit gene description	Codon sites examined	Sd	Nd	dS	dN
SINVm1_gene_07695	XM_011169556.1	Ornithine mitochondrial	274	1	3	0.008	0.007
SINVm1_gene_02125	NA	NA	88	0	1	0.000	0.006
SINVm1_gene_14492	NA	**Glucose dehydrogenase[1]**	**365**	**1**	**3**	**0.008**	**0.006**
SINVm1_gene_02111	NA	**Odorant binding protein SiOBP12[1] (Gotzek et al 2011)**	79	0	1	0.000	0.005
SINVm1_gene_07421	NA	Protein phosphatase 2C	93	0	1	0.000	0.004
SINVm1_gene_11240	NA	NA	116	0	1	0.000	0.004
SINVm1_gene_05180	NA	NA	158	0	1	0.000	0.004
SINVm1_gene_02113	XM_011159982.1	Trypsin	364	1	2	0.007	0.003
SINVm1_gene_05369	XR_850613.1	NA	135	0	1	0.000	0.003
SiOR91	NA	Odorant receptor SiOR91	273	0	1	0.000	0.002
SINVm1_gene_04163	XM_011161363.1	Mitochondrial RNA splicing protein	272	1	1	0.008	0.002
SINVm1_gene_12444	XM_011166400.1	N-acetyl-D-glucosamine kinase	305	1	1	0.008	0.002
SINVm1_gene_12447	XM_011166418.1	Dehydrogenase/reductase SDR member 11	224	0	1	0.000	0.002
SINVm1_gene_02165	XM_011176139.1	E3 ubiquitin-protein ligase	282	1	1	0.007	0.002
SINVm1_gene_11254	XM_011176174.1	NA	358	0	1	0.000	0.002
SINVm1_gene_02092	XM_011157589.1	NA	438	1	1	0.007	0.002
SINVm1_gene_11267	XM_011159571.1	SEC23-interacting protein	993	0	3	0.000	0.001
SiOR80	NA	**Odorant receptor SiOR80[1]**	329	2	1	0.010	0.001
SINVm1_gene_07708	XM_011169588.1	**Heparanase precursor[1]**	2138	4	4	0.005	0.001
SINVm1_gene_06259	XM_011166315.1	NA	1027	0	2	0.000	0.001
SINVm1_gene_04154	XM_011176993.1	**Glutamate receptor delta-2 subunit[1]**	564	1	1	0.004	0.001
SINVm1_gene_04177	XM_011171802.1	mRNA-decapping enzyme 2	560	2	1	0.009	0.001
SINVm1_gene_02105	XM_011176001.1	Methionyl-tRNA mitochondrial	648	3	1	0.011	0.001
SINVm1_gene_05331	XM_011158477.1	Molybdenum cofactor sulfurase	669	0	1	0.000	0.001
SINVm1_gene_02099	XM_011176044.1	Wolfram syndrome 1	670	1	1	0.003	0.001
SINVm1_gene_06270	XM_011166334.1	Growth arrest-specific protein 8	684	1	1	0.003	0.001
SINVm1_gene_11872	XM_011161398.1	Biorientation of chromosomes in cell division protein 1	825	1	1	0.003	0.001
SINVm1_gene_06284	XM_011166421.1	cGMP-specific 3',5'-cyclic phosphodiesterase	917	3	1	0.006	0.001
SINVm1_gene_14511	XM_011158412.1	FAM193A protein	1123	1	1	0.002	0.001

SINVm1_gene_12180	XM_011167108.1	Dynein heavy chain, axonemal	2898	10	3	0.006	0.000
SINVm1_gene_02187	XM_011175904.1	Centrosome-associated protein 350	2036	0	1	0.000	0.000
SINVm1_gene_14879	XM_011169573.1	Serine/threonine-protein kinase WNK	1835	2	1	0.002	0.000
SINVm1_gene_14488	XM_011166741.1	Vacuolar protein sorting-associated protein 13	2407	6	1	0.005	0.000

Table S1b: Sb haplotype

Local gene ID (assembly Si_gnH)	NCBI accession number (assembly Si_gnG)	Top blast hit gene description	Codon sites examined	Sd	Nd	dS	dN
SINVm1_gene_11877	XM_011171793.1	Sodium/potassium/calcium exchanger	73	0	2	0.000	0.012
SINVm1_gene_02092	XM_011157589.1	NA	438	0	5	0.000	0.008
SINVm1_gene_06282	XM_011166402.1	Nuclear hormone receptor HR96	385	3	5	0.020	0.007
SINVm1_gene_14492	NA	**Glucose dehydrogenase[1]**	**365**	**1**	**4**	**0.008**	**0.007**
SINVm1_gene_06964	XM_011161375.1	Ejaculatory bulb-specific protein 3	61	0	1	0.000	0.007
SINVm1_gene_05174	XM_011166958.1	Octopamine receptor	269	0	4	0.000	0.006
SINVm1_gene_12215	XM_011158399.1	NA	78	0	1	0.000	0.006
SINVm1_gene_14330	NA	NA	71	0	1	0.000	0.006
SINVm1_gene_04202	NA	NA	72	0	1	0.000	0.006
SINVm1_gene_02111	NA	**Odorant binding protein SiOBP12[1] (Gotzek et al 2011)**	79	0	1	0.000	0.005
SiOR84	XM_011176226.1	Odorant receptor SiOR84	286	0	2	0.000	0.005
SINVm1_gene_06290	XM_011166301.1	Retinol dehydrogenase	238	0	2	0.000	0.005
SINVm1_gene_07695	XM_011169556.1	Ornithine mitochondrial	274	0	2	0.000	0.005
SINVm1_gene_07421	NA	NA	93	0	1	0.000	0.004
SINVm1_gene_02200	XM_011159993.1	NA	304	1	3	0.005	0.004
SINVm1_gene_04189	XM_011171828.1	39S ribosomal protein mitochondrial	129	2	1	0.030	0.004
SINVm1_gene_06257	XM_011166415.1	Golgin subfamily A member 7	139	0	1	0.000	0.004
SINVm1_gene_04217	XM_011171736.1	Tubby-related protein 4	106	1	1	0.015	0.004
SINVm1_gene_05140	XM_011167170.1	Cuticle protein 6	284	0	2	0.000	0.004
SINVm1_gene_05157	XM_011166764.1	H/ACA ribonucleoprotein complex subunit 2	132	0	1	0.000	0.004
SINVm1_gene_11240		NA	116	0	1	0.000	0.004
SiOR95	XM_011176211.1	Odorant receptor SiOR95	222	0	1	0.000	0.003
SINVm1_gene_04176	XM_011171794.1	Tetratricopeptide repeat protein 1	178	0	1	0.000	0.003
SiOR93		Odorant receptor SiOR93	237	1	1	0.014	0.003
SINVm1_gene_11876	XM_011171849.1	Nicotinamide mononucleotide adenylyltransferase 1	190	0	1	0.000	0.003
SINVm1_gene_04158	XM_011176859.1	Peroxisomal membrane protein 11C	168	0	1	0.000	0.003
SINVm1_gene_11254	XM_011176174.1	NA	358	1	2	0.007	0.003
SINVm1_gene_06287	XM_011166404.1	Glutaredoxin 3	180	0	1	0.000	0.003
SINVm1_gene_04198	XM_011176912.1	Transmembrane protein 194A	189	1	1	0.013	0.003
SINVm1_gene_02199	XM_011159971.1	Slit protein	458	2	3	0.006	0.003
SINVm1_gene_04216	XM_011161415.1	Glycerol-3-phosphate acyltransferase mitochondrial	635	0	3	0.000	0.003

SINVm1_gene_02152	XM_011159856.1	RNA/RNP complex-1-interacting phosphatase	164	0	1	0.000	0.003
SiOR87	NA	Odorant receptor SiOR87	250	0	1	0.000	0.003
SINVm1_gene_05321	XM_011158424.1	39S ribosomal protein mitochondrial	263	0	1	0.000	0.002
SINVm1_gene_07708	XM_011169588.1	**Heparanase precursor**[1]	2138	4	8	0.005	0.002
SINVm1_gene_04214	XM_011161200.1	Receptor expression-enhancing protein 1	587	1	2	0.004	0.002
SINVm1_gene_04177	XM_011171802.1	mRNA-decapping enzyme 2	560	0	2	0.000	0.002
SINVm1_gene_12444	XM_011166400.1	N-acetyl-D-glucosamine kinase	305	1	1	0.008	0.002
SINVm1_gene_06275	XM_011166324.1	WD repeat-containing protein 91	561	0	2	0.000	0.002
SiOR97	XM_011176198.1	Odorant receptor SiOR97	314	1	1	0.000	0.002
SINVm1_gene_06278	XM_011166311.1	X-ray repair cross-complementing protein 5	336	0	1	0.000	0.002
SiOR96	XM_011176205.1	Odorant receptor SiOR96	343	1	1	0.006	0.002
SINVm1_gene_04228	XM_011171814.1	ef-hand domain-containing protein kiaa0494	343	0	1	0.000	0.002
SINVm1_gene_12222	XM_011158455.1	Disulfide-isomerase a4 protein	362	1	1	0.005	0.002
SINVm1_gene_05360	XM_011158446.1	KAT8 regulatory NSL complex subunit 3 isoform x5	866	1	2	0.003	0.001
SINVm1_gene_11247	NA	Transcription factor collier	325	1	1	0.004	0.001
SINVm1_gene_11267	XM_011159571.1	SEC-23 interacting protein p125	993	5	3	0.008	0.001
SiOR80	NA	**Odorant receptor SiOR80**[1]	329	3	1	0.015	0.001
SINVm1_gene_02183	XM_011175944.1	Tubulin alpha chain	352	0	1	0.000	0.001
SINVm1_gene_06268	XM_011166349.1	Erythroid differentiation-related factor 1	926	1	2	0.003	0.001
SINVm1_gene_11263	XM_011159458.1	Slit protein	395	1	1	0.004	0.001
SINVm1_gene_06283	XM_011166398.1	Choline ethanolaminephosphotransferase 1-like isoform 2	430	1	1	0.004	0.001
SINVm1_gene_07701	XM_011163661.1	Tubulin polyglutamylase ttll9	372	0	1	0.000	0.001
SINVm1_gene_04154	XM_011176993.1	**Glutamate receptor delta-2 subunit**[1]	564	3	1	0.013	0.001
SINVm1_gene_04230	XM_011171807.1	Calcium-independent phospholipase a2-gamma	485	1	1	0.004	0.001
SINVm1_gene_05177	XM_011171493.1	Glucosyl glucuronosyl transferases	372	0	1	0.000	0.001
SINVm1_gene_09040	XM_011157222.1	Maltase 1	501	0	1	0.000	0.001
SINVm1_gene_06276	XM_011166322.1	T family of potassium channels protein 18	560	0	1	0.000	0.001
SINVm1_gene_12223	XM_011158466.1	Maltase 1	408	0	1	0.000	0.001
SINVm1_gene_14335	XM_011171809.1	Atrial natriuretic peptide receptor 1	559	1	1	0.003	0.001
SINVm1_gene_11875	XM_011171849.1	Nicotinamide mononucleotide adenylyltransferase 1	647	0	1	0.000	0.001
SINVm1_gene_06269	XM_011166331.1	Cytoplasmic aconitate hydratase	962	4	2	0.006	0.001
SINVm1_gene_11874	XM_011171736.1	Tubby-related protein 4	977	4	2	0.006	0.001

SINVm1_gene_04179	XM_011171825.1	Sorting nexin-27	1465	6	3	0.006	0.001
SINVm1_gene_02160	XM_011157595.1	Leishmanolysin-like peptidase	536	1	1	0.003	0.001
SINVm1_gene_02105	XM_011176001.1	Methionyl-tRNA, mitochondrial	648	3	1	0.011	0.001
SINVm1_gene_05355	XM_011158426.1	Dipeptidyl peptidase 9	657	1	1	0.003	0.001
SINVm1_gene_02099	XM_011176044.1	Wolframin	670	1	1	0.003	0.001
SINVm1_gene_13971	XM_011159701.1	U4/U6 small nuclear ribonucleoprotein prp3	603	0	1	0.000	0.001
SINVm1_gene_06294	XM_011166388.1	Catenin alpha	719	3	1	0.009	0.001
SINVm1_gene_04170	XM_011171742.1	tRNA (guanine(26)-N(2))-dimethyltransferase (probable)	910	1	1	0.003	0.001
SINVm1_gene_13984	XM_011163055.1	Tyrosine-protein kinase transmembrane receptor ROR1	772	1	1	0.003	0.001
SINVm1_gene_02087	XM_011159713.1	Heat shock 70 kda protein cognate 5	777	2	1	0.005	0.001
SINVm1_gene_02156	XM_011157586.1	Endoplasmic reticulum aminopeptidase 2	829	2	1	0.005	0.001
SINVm1_gene_14883	XM_011163661.1	PH and SEC7 domain-containing protein 3	860	4	1	0.009	0.001
SINVm1_gene_02182	XM_011175961.1	Dynactin subunit 1	934	2	1	0.005	0.001
SINVm1_gene_06259	XM_011166315.1	flj37770-like protein	1027	1	1	0.002	0.001
SINVm1_gene_05143	XM_011167027.1	Phosphatidylinositide phosphatase sac2	933	0	1	0.000	0.001
SINVm1_gene_11880	XM_011171791.1	Huntingtin	2253	11	3	0.008	0.001
SINVm1_gene_06260	XM_011166304.1	Melanotransferrin	1085	0	1	0.000	0.001
SINVm1_gene_06284	XM_011166421.1	cGMP-specific 3 -cyclic phosphodiesterase	917	5	1	0.010	0.001
SINVm1_gene_11869	XM_011171733.1	DE-cadherin-like isoform x4	1250	2	1	0.004	0.000
SINVm1_gene_14519	XM_011158437.1	Exportin-7 isoform x1	1040	0	1	0.000	0.000
SINVm1_gene_14488	XM_011166741.1	Vacuolar protein sorting-associated protein 13b	2407	0	2	0.000	0.000
SINVm1_gene_12180	XM_011167108.1	Dynein heavy chain axonemal	2898	6	3	0.003	0.000
SINVm1_gene_04159	XM_011176848.1	Cell division cycle protein 16 homolog	1008	3	1	0.005	0.000
SINVm1_gene_02121	XM_011163067.1	Transcription elongation factor SPT5	1221	2	1	0.002	0.000
SINVm1_gene_05368	XM_011158467.1	Chaoptin	1252	1	1	0.001	0.000

Local gene ID: identifier for newly annotated or re-annotated genes (for which NCBI accessions are given); all sequences are available in Supplementary Files S1 and S2.

Sd: number of synonymous differences; Nd: number of nonsynonymous differences; dS: synonymous substitutions per synonymous site; dN: nonsynonymous substitutions per nonsynonymous site.

[1] Appears in both SB and Sb lists.
