# Supplementary material for: Evolution of Olfactory Functions on the Fire Ant Social Chromosome
Source: Genome Biol Evol. 2018 Sep 18;10(11):2947–60. doi: 10.1093/gbe/evy204 (PMC6279166; doi:10.1093/gbe/evy204)
Supplement: Supplementary Data [file evy204_supp.zip › Table S2.OR polymorphism.pdf]

Table S2: Divergence and polymorphism in the cluster of *S. invicta* OR genes

	S. invicta					*SB*				*Sb*			
Gene	Ps	Pn	Fs	Fn	Branch-site test q-value	Ps	Pn	Fs	Fn	Ps	Pn	Fs	Fn
SiOR76	6	5	19	13	0.884	2	3	0	0	0	0	3	1
SiOR77	3	4	6	25	0.535	2	2	1	0	0	0	1	1
SiOR78	4	6	16	21	0.064	0	3	0	0	0	1	4	2
SiOR79	0	0	26	42	0.884	0	0	0	0	0	0	0	0
SiOR80	6	3	16	3	0.884	2	0	1	1	0	0	2	1
SiOR81	15	14	4	2	0.884	12	14	5	5	0	1	1	1
SiOR82	2	7	8	17	0.979	2	3	0	0	0	1	0	0
SiOR83	7	1	2	0	0.884	5	1	0	0	0	0	1	0
SiOR84	3	5	14	10	0.608	3	3	2	2	0	0	0	2
SiOR85	8	0	19	12	0.884	3	0	1	0	0	0	4	0
SiOR86	3	8	3	4	0.884	2	5	0	1	0	0	0	0
**SiOR87**	6	2	10	20	0.053	**4**	**0**	**2**	**0**	**1**	**0**	**1**	**3**
SiOR88	1	3	2	5	0.324	1	3	0	0				
SiOR89	0	0	17	16	0.111	0	1	0	0				
SiOR90	5	4	7	4	0.884	3	0	2	1	0	0	2	2
SiOR91	2	1	11	7	0.884	1	1	1	1	0	0	0	0
SiOR92	4	4	13	9	0.884	4	3	1	1	0	0	0	1
SiOR93	7	2	12	17	0.884	0	0	2	1	0	0	1	1
SiOR94	2	2	10	14	0.884	1	0	0	0	0	0	1	0
SiOR95	2	4	11	18	0.801	0	0	0	0	0	0	0	1
SiOR96	1	2	12	8	0.324	1	0	0	0	0	0	0	0
SiOR97	1	4	14	8	0.884	1	3	0	0	0	0	0	0
SiOR98	3	3	13	7	0.884	1	1	0	1	0	0	1	1

Ps, Pn are the number of synonymous and nonsynonymous polymorphic sites respectively; Fs, Fn are the number of synonymous and nonsynonymous substitutions respectively. Counts under *S. invicta* summarize polymorphism in all *S. invcta* genotypes and substitutions along the *S. invicta* branch prior to the divergence between SB and Sb. Counts under SB and Sb are limited to polymorphism within each haplotype group and substitutions that occurred in each branch after their divergence. The branch-site test *q*-value is based on the likelihood ratio test.
