# Supplementary material for: Evolution of Olfactory Functions on the Fire Ant Social Chromosome
Source: Genome Biol Evol. 2018 Sep 18;10(11):2947–60. doi: 10.1093/gbe/evy204 (PMC6279166; doi:10.1093/gbe/evy204)
Supplement: Supplementary Data [file evy204_supp.zip › Table S3.primers.pdf]

Table S3: primer list

Purpose	Name	F-primer	R-primer	Length (DNA/mRNA)	Target
Gp-9 genotyping[51]	24bS, 25bAS	TGGAGCTGATTATGATGAAGAGAAAATA	GCTGTTTTTAATTGCATTTCTTATGCAG	423	Sb
	26BS, 16BAS	CTCGCCGATTCTAACGAAGGA	ATGTATACTTTAAAGCATTCCTAATATTTTGTC	517	SB
Validation of deletion	Deletion_545	ATGCCCTGAGGCTAGAGGAA	GCGATAGGAAAGGCAGGGTT	545	SB&Sb
	Deletion_875	TGTACAGGAACAAGAGTGACAAG	AGCACTTAATTGAAACCCACAG	875	Sb
HKG	rpl_18	CCTGGAAGGGAGAATTGCGT	TTTCAGGATACGTGCCCGAG	129/129	SB&Sb
	ef1_beta	TGGGGAAACCACCAACACAA	GGTGCCAAAGATGGCAGGTA	122/122	SB&Sb
SiOR genes	OR86	CCATCAACGTGCCTGTAGGA	TCAAACTGACCGCAAAGATGC	149/149	SB&Sb
	OR87	GGCCCCTACAAAAGAAGACGA	TAGACACCACGCATACAGCG	383/133	SB&Sb
	OR88	AATGCTTTGCATCCGCCTTC	GACCGTAGAGATGCACAGTGA	345/142	SB
	OR89	GGTGACACCGGTAGAACCAT	ATCGTCGGAAGCAGCATTGA	129/129	SB
