# Supplementary material for: Evolution of Olfactory Functions on the Fire Ant Social Chromosome
Source: Genome Biol Evol. 2018 Sep 18;10(11):2947–60. doi: 10.1093/gbe/evy204 (PMC6279166; doi:10.1093/gbe/evy204)
Supplement: Supplementary Data [file evy204_supp.zip › Table S4. Binomial tests for positive selection enrichment in OR structural domains.pdf]

Supplementary Table S4: Binomial tests for positive selection enrichment in OR structural domains

Structure	region size	No. of positively selected sites	*p*-value	FDR corrected *q*-value
**-**	33	**1**	0.9447222	0.9999999
**TM1**	22	**0**	0.9999999	0.9999999
**EC1**	14	**0**	0.9999999	0.9999999
**TM2**	21	**3**	0.2538452	0.9519195
**IC1**	34	**2**	0.7944034	0.9999999
**TM3**	25	**5**	0.0570374	0.285187
**EC2**	25	**1**	0.8872535	0.9999999
**TM4**	25	**0**	0.9999999	0.9999999
**IC2**	60	**3**	0.8991254	0.9999999
**TM5**	26	**1**	0.8949377	0.9999999
**EC3**	6	**3**	0.0131647	0.09873525
**TM6**	23	**1**	0.860787	0.9999999
**IC3**	24	**10**	0.0000179	0.0002685
**TM7**	37	**3**	0.6110648	0.9999999
**-**	14	**1**	0.5359972	0.9999999
