# Supplementary material for: Evolution of Olfactory Functions on the Fire Ant Social Chromosome
Source: Genome Biol Evol. 2018 Sep 18;10(11):2947–60. doi: 10.1093/gbe/evy204 (PMC6279166; doi:10.1093/gbe/evy204)
Supplement: Supplementary Data [file evy204_supp.zip › Table S5.Conservation of amino-acid differences between the SB and Sb haplotypes.pdf]

Table S5: Conservative and non-conservative amino-acid differences between the SB and Sb haplotypes

Position (amino-acid)	OR-ID	Variant	Conservative
42	SiOR95	A42G	yes
43	SiOR95	M43I	yes
77	SiOR95	A77V	yes
100	SiOR95	A100V	yes
111	SiOR78	K111E	yes
116	SiOR78	I116N	no
124	SiOR92	S124T	yes
125	SiOR78	R125K	yes
128	SiOR86	F128L	yes
129	SiOR94	N129D	yes
141	SiOR87	L141F	yes
172	SiOR76	T172I	no
181	SiOR98	S181W	no
186	SiOR90	C186Y	no
199	SiOR96	S199L	no
233	SiOR94	T233I	no
234	SiOR84	S234N	yes
236	SiOR77	N236K	yes
243	SiOR97	M243I	yes
319	SiOR87	R319G	no
331	SiOR93	T331I	no
332	SiOR80	N332D	yes
338	SiOR77	A338V	yes
340	SiOR98	R340I	no
348	SiOR76	I348T	no
351	SiOR84	S351L	no

Highlighted positions are in the IC3 domain
